# Potential for synergistic conservation through area‐based strategies

**DOI:** 10.1111/cobi.14447

**Published:** 2025-02-13

**Authors:** Li Zhang, Yanwen Fu, Jiaxin Li, Lingyan Yan, Xiaojun Kou, Zhiyun Ouyang

**Affiliations:** ^1^ State Key Laboratory of Urban and Regional Ecology, Research Center for Eco‐Environmental Sciences Chinese Academy of Sciences Beijing China; ^2^ College of Life Sciences Beijing Normal University Beijing China; ^3^ College of Fisheries and Life Science Dalian Ocean University Dalian China

**Keywords:** biodiversity, decoupling, fish, spatial patterns, species–area relationships, biodiversidad, desvinculación, patrones espaciales, peces, relación especie‐área

## Abstract

The ongoing biodiversity crisis has raised concerns about the effectiveness of area‐based conservation (ABC) strategies for achieving positive biodiversity outcomes. In riverine ecosystems, the linear habitat structure of fishes introduces uncertainty into the synergistic conservation potential of ABC. Therefore, to assess the synergistic conservation potential of ABC for multiple groups, we used data from IUCN and RivFishTIME database up to 2020 for fishes, mammals, and birds to assess the reliability of area sampling based on species–area relationships and latitudinal dependence analyses. We built a spatial model of species richness to determine the spatial distribution of species richness and the spatial overlap of species richness within and among the 3 groups under different group combinations. We found a significant power function relationship between area and species richness; *R* ^2^ values ranged from 0.94 to 0.96. Species richness was unevenly distributed across groups; thus, the potential for synergistic conservation of multiple groups is not promising. Fish were outliers. The spatial overlap for fish–bird combinations (*β* = −0.001 to 0.02) and fish–mammal combinations (*β* = 0.10–0.11) were significantly lower than those for mammal–bird combinations (*β* = 0.20−0.27). This calls for targeted conservation planning for fishes in terrestrial ecosystems rather than considering that protected areas for mammals and birds will also protect fishes. Furthermore, the synergistic conservation potential of multitarget strategies cannot be safely extended to all groups.

## Abstract (es)

Potencial para la conservación sinérgica por medio de estrategias basadas en el área

**Resumen:**

La actual crisis de la biodiversidad genera inquietud sobre la eficacia de las estrategias de conservación basadas en el área (CBA) para lograr resultados positivos para la biodiversidad. En los ecosistemas fluviales, la estructura lineal del hábitat de los peces introduce incertidumbre en el potencial sinérgico de conservación de la CBA. Por lo tanto, para evaluar el potencial de conservación sinérgico de la CBA para múltiples grupos, utilizamos datos de la UICN y de la base de datos RivFishTIME hasta 2020 para peces, mamíferos y aves con el fin de evaluar la fiabilidad del muestreo de áreas basado en las relaciones especie‐área y los análisis de dependencia latitudinal. Construimos un modelo espacial de riqueza de especies para determinar la distribución espacial de la riqueza de especies y el traslape espacial de la riqueza de especies dentro de los tres grupos y entre ellos bajo diferentes combinaciones de grupos. Encontramos una relación significativa de función de potencia entre el área y la riqueza de especies; los valores de R^2^ oscilaron entre 0.94 y 0.96. La riqueza de especies se distribuyó desigualmente entre los tres grupos, por lo que el potencial de conservación sinérgica de múltiples grupos no es prometedor. Los peces fueron valores atípicos. El traslape espacial de las combinaciones pez‐ave (β = ‐0.001 a 0.02) y pez‐mamífero (β = 0.10‐0.11) fueron significativamente menores que el de las combinaciones mamífero‐ave (β = 0.20‐0.27). Esto exige una planificación de la conservación específica para los peces en los ecosistemas terrestres en lugar de considerar que las áreas protegidas para mamíferos y aves también protegerán a los peces. Además, el potencial sinérgico de conservación de las estrategias con múltiples objetivos no puede extenderse con seguridad a todos los grupos.

## INTRODUCTION

The biodiversity crisis continues (Díaz et al., 2019; Myers et al., 2000; Rockström et al., 2009), raising concerns about the effectiveness of area‐based conservation (ABC) strategies in halting biodiversity loss (Maxwell et al., 2020; Meng et al., 2023). Although ABC strategies are considered cornerstones of biodiversity conservation (Dobrowski et al., 2021), improving their synergistic conservation potential is critical to the successful implementation of ABC strategies and to fostering public trust in conservation efforts.

The spatial distribution of different groups may affect the synergistic conservation potential of ABC strategies. In ABC, it is ideal for a protected area to benefit more than one species or group of species to provide conservation synergism (Mi et al., 2023; Shen et al., 2023). However, the different responses of different groups to biotic and abiotic factors may result in a heterogeneous distribution of species in a landscape, which makes spatial planning difficult (Blowes et al., 2019; Pianka, 1966). For example, in the context of global change, amphibians have a higher probability of being threatened than mammals and birds (Harfoot et al., 2021). The spatial heterogeneity of climatic geography further complicates determining biodiversity spatial patterns among different groups (Coelho et al., 2023). However, the effect of differences in biodiversity spatial patterns among different groups on the synergistic conservation potential of ABC is largely unknown.

Fish have not received sufficient attention in the study of biodiversity spatial patterns. Freshwater fishes depend on rivers or lakes for survival and are facing a greater potential for extinction than terrestrial and marine species (Ward, 1998). According to the Living Planet Index, humans have more of a negative impact on freshwater vertebrates than on terrestrial or marine vertebrates (83% reduction in freshwater species and 40% reduction in other species) (Reid et al., 2019; UNEP‐WCMC & IUCN, 2016). Fish make up one third of freshwater vertebrates and are one of the most endangered vertebrate groups (Allan & Flecker, 1993; Balian et al., 2008). Despite their facing a high threat level, freshwater ecosystems are often confused with other terrestrial landscapes (Heilpern, 2015; McFadden et al., 2023). Some researchers are stressing the importance of conserving freshwater fishes (Fausch et al., 2002; Leal et al., 2020), but their habitat structure makes it difficult to explore synergistic conservation that includes (Blowes et al., 2019) other terrestrial groups.

In terrestrial ecosystems, insufficient attention has been given to the linear habitat structure of fishes when analyzing their distributional patterns (Barnosky et al., 2011; Mi et al., 2023). For example, some studies have examined changes in temporal patterns of different groups in river basins (Blowes et al., 2019; Su et al., 2021). Temporal changes in biodiversity may be influenced greatly by the timing of sampling. Therefore, one must ensure that the size of the areas sampled is consistent among sampling events. When conducting spatial analyses, one needs to determine species richness in a sampling unit, which needs to ensure that the sampling unit is sampled in the same way. However, for mammals and birds, habitat can be considered in area units, but for rivers, it is more common to consider habitat in linear units. It is unclear whether rivers can be effectively considered in area units. The discrepancy between area units and linear units may introduce uncertainty into the results of studies on spatial patterns of biodiversity. Uncertainty thus becomes part of evaluations of the synergistic conservation potential of ABC.

We used consistent area sampling to improve the robustness of the examination of the spatial distribution of species richness in fishes, mammals, and birds (Yanwen et al., 2023). Consistent area sampling means that the species richness of fishes, mammals, and birds was measured in units of area. We then assessed the appropriateness of area sampling for different groups by analyzing the species–area relationship (SAR) and latitude dependence, which reflect the spatial distribution patterns of species richness (Hawkins, 2001; Hillebrand, 2004). Finally, we conducted spatial modeling of species richness, identified the spatial distribution of areas with high and low species richness, and examined the overlap of species richness within and between the 3 groups under different group combinations.

## METHODS

### Data sources

We obtained species distribution data for fishes, birds, and mammals from publicly available databases, which were divided into 2 formats: global terrestrial raster data for mammals and birds and riverine point survey data for fishes. We obtained the raster data from a public data set published by Lumbierres et al. (2022), who mapped the area of habitat (AOH) for each species based on species distribution information from the International Union for Conservation of Nature (IUCN) Red List. The AOH is “the habitat available to a species, that is, the habitat within its range.” It is obtained by removing areas that are not habitat from the species range map, indicating the most likely occupied areas within the species range, thus reducing commission errors. The data included distribution information on migratory birds, whose habitat use changes over an annual cycle (Schuster et al., 2019). To obtain robust area sampling results, we used only data on nonmigratory birds. Ultimately, 5484 mammal species and 8835 bird species were included in our analyses. The fish data were compiled from independent sampling surveys on a global scale. The average survey duration was 8 years (Comte et al., 2021). We focused on spatial rather than temporal variation in species richness; therefore, we collected fish species records from each independent survey in the most recent year to examine the global riverine distribution of fish species (Figure 2a). We included 713 fish species in our analyses.

### Area sampling

**FIGURE 1:**
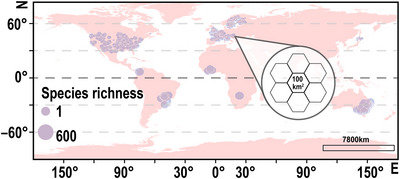
Global distribution of 1338 mammal, bird, and fish samples and number of unique species in each sample.

The spatial coverage of the data on the 3 groups was not uniform; fish data had the smallest spatial coverage. Therefore, we gridded the fish spatial extent with hexagons (100 km^2^, 1338 in total) (Galiana et al., 2022) (Figure 1) (Appendix S1). The hexagon size ensured it contained the necessary number of fish survey points for statistical analyses; this was the highest resolution in our species‐related analyses. When we directly mapped the species richness of the 3 groups on a hexagonal grid, we saw that sampling was not uniform. The fish data were organized by sampling location, similar to incomplete sampling, whereas the mammal and bird data were organized by the AOH, similar to complete sampling. To unify the sampling type, we marked 10 random points (based on the average sampling intensity of fishes: 8.5 times/hexagon) in each hexagon and extracted the mammal and bird species richness for each point separately (Appendix S2). Thus, we generated bird and mammal data organized by sampling location, as for the fish data. Finally, we extracted species names in the hexagon and removed duplicate species to obtain a unique species richness value for each hexagon (Appendices S3 & S5). The operation was completed using ArcGIS Pro 2.0 software (Environmental Systems Research Institute).

### Testing SARs and latitudinal dependence

To calculate the latitudinal distribution of species richness, we extracted the longitude and latitude corresponding to the hexagon centroid and combined different groups. Within the hexagons, we considered each group by itself and 2 and 3 groups combined. We then determined the species richness in the hexagon for each combination. Correlation analysis was used to examine the relationship between species richness and latitude among the combinations, and the results were tested by correlation (*r*) and the *p* value. Statistical analysis was performed using IBM SPSS software.

When calculating the SAR, we generated an area change gradient of 100–10,000 km^2^ by randomly combining different numbers of hexagons with a gradient interval of 100 km^2^ (Galiana et al., 2022). The species richness for each gradient was the number of species in each hexagon. For example, when the 1000‐km^2^ gradient was generated, we randomly selected 10 hexagons among all the hexagons to combine and noted the species names therein to determine the species richness corresponding to the 1000‐km^2^ gradient. Ten randomly selected hexagons could contain the same species; therefore, a deduplication operation was required to obtain an accurate species richness value. The geographical positions of these 10 hexagons were random, and to obtain a robust species richness distribution within the gradient, we repeated the above process 100 times and used the average species richness corresponding to the 1000‐km^2^ gradient. We applied the above processes to all 100 gradients. We used power functions to fit the SAR and calculated *p* and *R* ^2^ to evaluate the fit of our model (species richness = *k×*area^a^). We only analyzed the SAR within each group. The operation was conducted with Python 3.8 software.

To further verify the reliability of the random combination method, we extracted the length of the river contained in the hexagon (Lehner et al., 2008) and used the same method to determine the river length–area relationship (Appendix S6). To generate the area gradient, we used another method. We increased the area of the original hexagon, generated grids that covered the fish locations, determined the river length in the hexagons with different areas, from 10 to 500 km^2^, and tested the river length–area relationships again (Appendix S7). This operation was also conducted with Python 3.8 software.

### Spatial autocorrelation analyses within a group

We performed a Moran analysis on the hexagonal species richness data to determine the co‐occurrence of high species richness within a group. First, we constructed a spatial weight matrix (Appendix S8) for all the hexagons to describe the adjacency relationships between the hexagons (Anselin & Bera, 1998). The adjacency weight was 6 because there were at most 6 adjacent hexagons around a hexagon. Adjacency was marked as 1, and nonadjacency was marked as 0. Then, we used global Moran's *I* to determine whether the species richness within a group exhibited an aggregation trend. Finally, we used local Moran's *I* with the group that showed an aggregation trend. The species richness values of each hexagon and its adjacent hexagon were compared with those of all hexagons within the group, and the local co‐occurrence of high species richness was quantified with Moran's *I*. Moran's analysis was used to evaluate the efficiency of using *Z* scores and *p* values. The operation was based on GeoDa software (Anselin et al., 2006). See Appendices S9–S18 for a more detailed description of the methods used.

### Cross‐group spatial correlation analyses

To test the co‐occurrence of high species richness across groups, we used a spatial econometric model (Hao & Liu, 2016). Spatial econometric models for spatial autocorrelation could be considered extensions of ordinary least squares (OLS) regression models because they explicitly incorporate spatial effects (Hao & Liu, 2016). First, we constructed 3 models to describe the spatial correlation of species richness between different groups, namely, the fish–mammal, fish–bird, and mammal–fish groups. The spatial position of species richness was determined by the corresponding hexagon. Second, we conducted OLS regression analyses for each model and a Lagrange multiplier (LM) test or robust LM test to determine whether the spatial error effect or spatial lag effect was reasonable to include in the analyses. The LM and robust LM tests generated 2 types of statistics, LM lag and LM error, respectively. If neither of the 2 statistics was significant, the OLS analysis was reasonable. If the LM lag was significant, the spatial lag analysis was reasonable, and if the LM error was significant, the spatial error analysis was reasonable. If both were significant, both analyses were reasonable (Hao & Liu, 2016). The efficiency of the model was determined based on *p* and *R* ^2^ values. Based on the LM test and the robust LM test, we incorporated the spatial error effect into the analyses to capture the high‐value co‐occurrence patterns between taxonomic groups (details in Appendices S21–S29).

### Difference analyses

To compare the differences in the degree of co‐occurrence of high species richness among different models, we set up 5 hexagonal area gradients ranging from 100 to 500 km^2^, with gradient intervals of 100 km^2^, and repeated the above area sampling method and cross‐group spatial correlation analyses for each gradient. From this, we obtained 5 replicates for the fish–mammal, fish–bird, and mammal–bird models. The differences between the independent variable (species richness of different groups) coefficients of the 3 models and *R* ^2^ values were compared. The operation was conducted with GeoDa (Anselin et al., 2006) and IBM SPSS software. Refer to Appendices S9–S18 for a more detailed description of the methods.

## RESULTS

**FIGURE 2:**
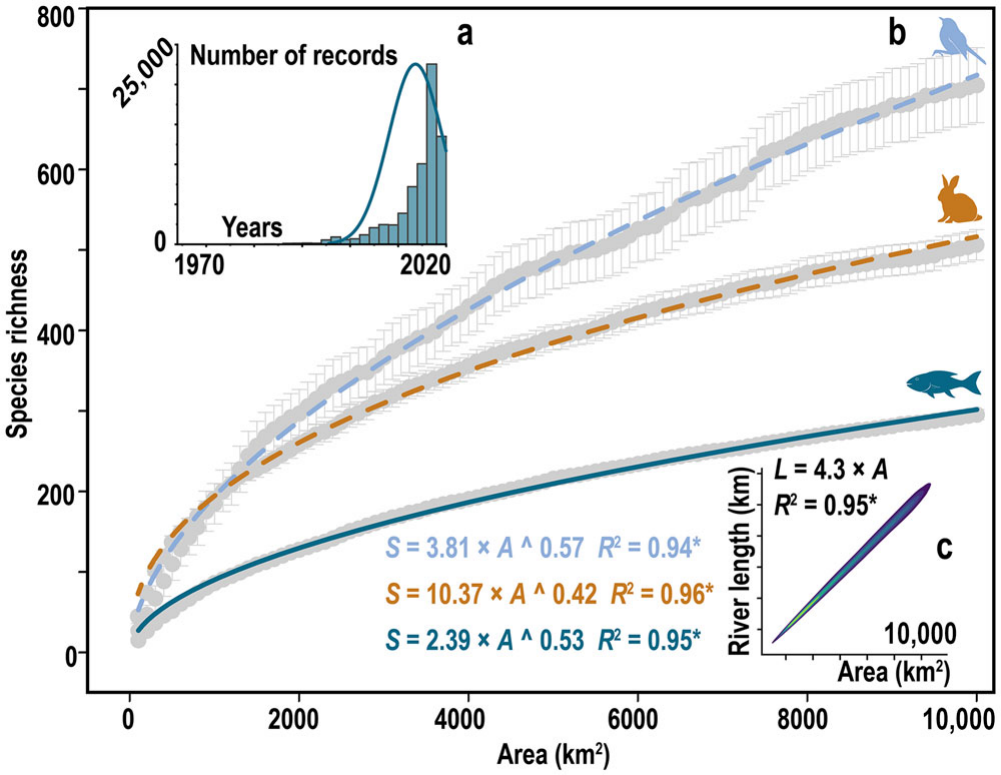
Changes in species richness and river length as area increases: (a) temporal distribution of recorded fish locations used for area‐sampling conversion, (b) global species–area curves for fishes, mammals, and birds, and (c) relationship between the sampling area size (*A*) and river length (*L*) based on random combination sampling (*p < 0.05).

The SAR had a significant power function trend (*p* < 0.05, *R* ^2^ = 0.94–0.96) in all 3 groups (Figure 2b). Although the fishes also inhabited linear regions rather than area regions, such as rivers, similar SARs still applied, which led us to investigate the relationship between area and river length.

River length in the sampled area increased linearly (*p* < 0.05, *R* ^2^ = 0.95–0.96) as the area increased, regardless of whether it was based on random sample combinations or the actual expansion gradient of the sampling area (10–500 km^2^) (Figure 2; Appendix S19). River length increased at a rate of 4.3 (slope) under both models of area expansion (Figure 2c; Appendix S19).

**FIGURE 3:**
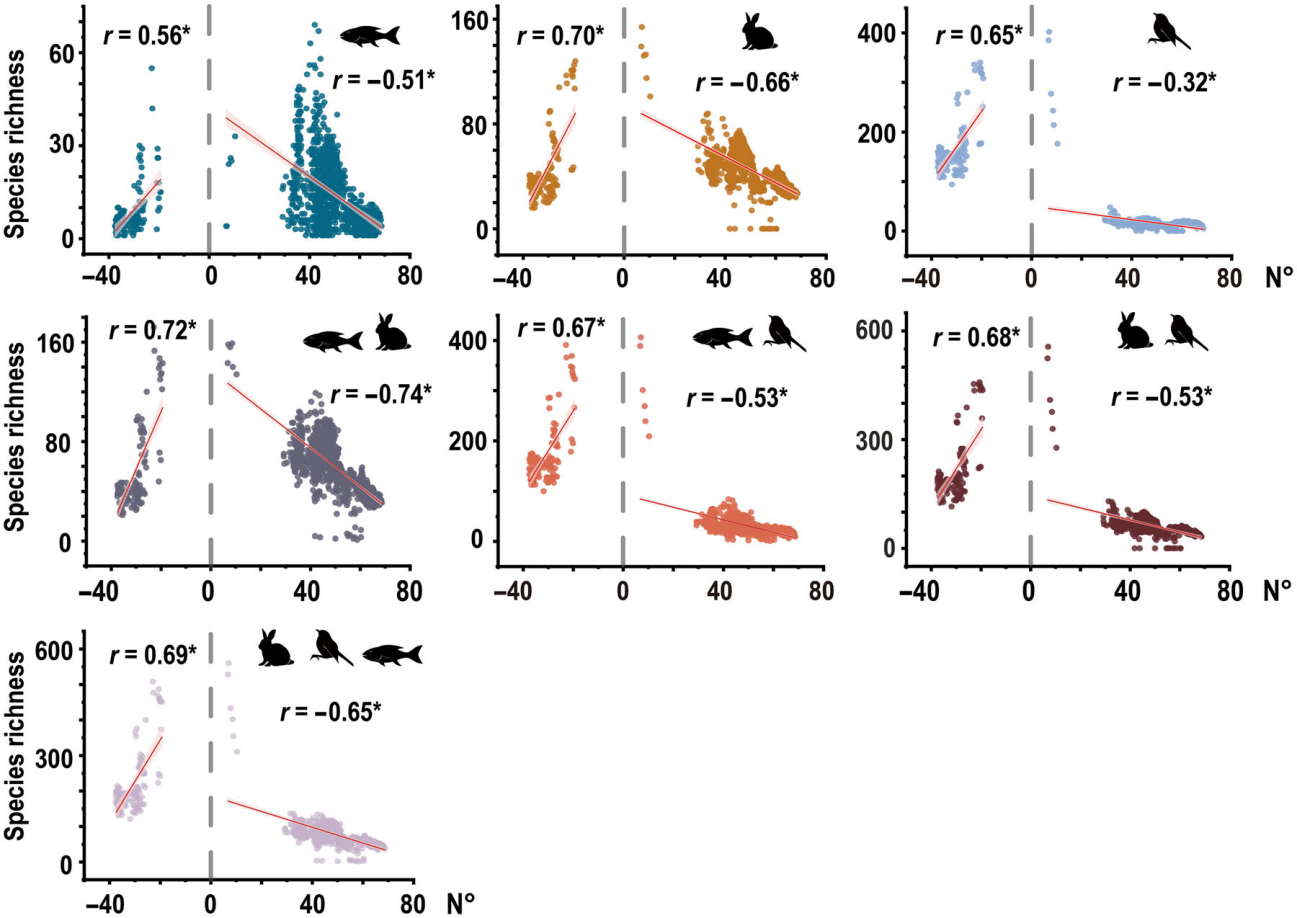
Latitudinal distribution of species richness for different combinations of fishes, birds, and mammals (vertical dashed line, equator; *p < 0.05).

Species richness distribution showed a consistent unimodal pattern relative to latitude—that is, species richness tended to decrease from the equator toward the poles (*r* = −0.32 to 0.72, *p* < 0.05) (Figure 3). This pattern was observed across different groups and their combinations, demonstrating the appropriateness of area sampling.

**FIGURE 4:**
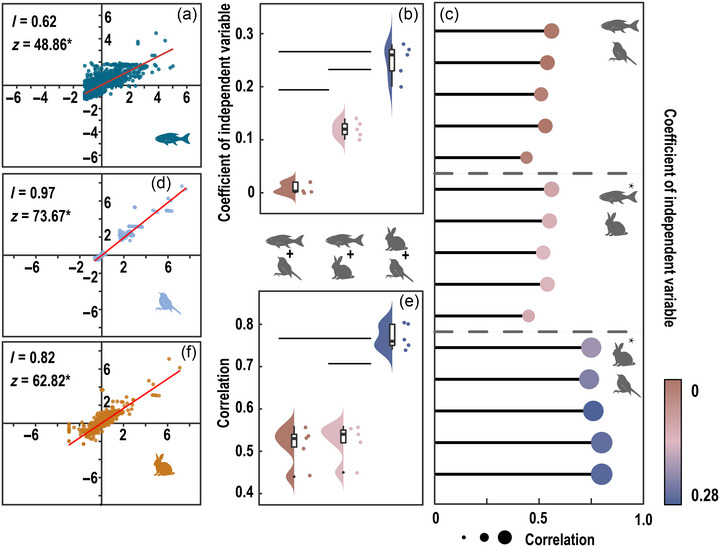
Co‐occurrence of high species richness within and among fishes, birds, and mammals: (a, d, f) spatial aggregation trend of species richness within groups, (b) differences in the degree of protection synergism (independent variable, species richness of different groups) among different groups (lines, significant differences between 2 combinations [*p* < 0.05]), (c) spatial trend of high species richness among the 3 groups (bars from top to bottom, 5 replicates from 100 to 500 km^2^ in the combinations; line length, relative *R* ^2^ value; color gradient, slope, which indicates the degree of synergism), and (e) spatial correlation differences in species richness among groups.

Based on the spatial weight matrix, we identified 86 hexagons (sampling units) as having no neighbors and subsequently excluded them from the analyses, approximately 6% of all hexagons (Appendix S8). Within each taxonomic group, we observed a significant spatial aggregation trend in species richness, suggesting that species tended to cluster in certain geographic locations. The global *I* values, which measure the degree of spatial aggregation, were 0.62 for fishes, 0.97 for birds, and 0.82 for mammals (Figure 4a,d,f). The local *I* analyses indicated that synergistic aggregation, where sample units with high and low species richness co‐occurred, was prevalent. Specifically, synergistic aggregation occurred in 66% of the mammals, 79% of the birds, and 60% of fishes (Appendix S20).

Among the analyzed groups, we observed a significant trend of co‐occurrence of high species richness between mammals and birds, as indicated by *R* ^2^ values ranging from 0.75 to 0.80 (*p* < 0.05) (Figure 4c; Appendix S24). Furthermore, the synergism between mammals and birds (*β* = 0.20–0.27, *p* < 0.05) was significantly greater than the synergism between fishes and mammals (*β* = 0.10–0.11, *p* < 0.05) or between fishes and birds (*β* = −0.001 to 0.02, *p* > 0.05) (Figure 4e; Appendices S25 & S26). We observed a significant trend toward co‐occurrence between fishes and mammals (*R* ^2^ = 0.45–0.56, *p* < 0.05) (Figure 4c; Appendix S25). However, the synergism between fishes and mammals was significantly lower than that between mammals and birds (Figures 3e & 4b). In contrast, we did not observe a significant co‐occurrence of high species richness between fishes and birds (*R* ^2^ = 0.44–0.56, *p* > 0.05) (Figure 4c; Appendix S26).

## DISCUSSION

In the context of the global biodiversity crisis, assessing the synergistic conservation potential of ABC can help achieve positive biodiversity outcomes and enhance the social credibility of conservation efforts. We analyzed co‐occurrence trends in the species richness of fishes, mammals, and birds in terrestrial ecosystems. Our aim was to determine the synergistic conservation potential of ABC strategies and to test the rationality of area sampling in riverine structures for extracting species richness. Our results indicated that fishes conform to the SAR and latitude dependence, suggesting that area sampling is a reasonable approach for studying species richness across groups on a global scale. Furthermore, different combinations of species groups corresponded to distinct co‐occurrences of high species richness. This observation suggests that the synergistic conservation potential of ABC in multigroup scenarios is not promising and therefore requires careful implementation.

The SAR of fishes provided theoretical support for the application of ABC in freshwater ecosystems. As biodiversity loss continues, ABC could be beneficial for the conservation of freshwater ecosystems because rivers are influenced by the surrounding terrestrial landscape (Dudgeon et al., 2006; Tickner et al., 2020). Our results showed that fish species richness also followed the SAR on a global scale. Although SARs for mammals and birds are well documented and used to predict extinction probabilities or guide ABC (Arrhenius, 1921; He & Hubbell, 2011; Pereira et al., 2010), few studies have reported or tested the validity of SARs for fishes on a global scale (Blowes et al., 2019; Heilpern, 2015). Fishes depend on freshwater ecosystems for their survival, and although lakes can be treated as areal units, rivers exist as linear units (Carraro & Altermatt, 2022; Grill et al., 2019). Some studies have defined river networks as dendritic networks and investigated the effects of network topology on ecological processes, emphasizing the importance of the mixed effects of catchment area and network structure complexity in increasing diversity on a regional scale (Campbell Grant et al., 2007; Larsen et al., 2021; Terui et al., 2021). However, focusing only on linear river management on a regional scale may not effectively address the threats to freshwater ecosystems in the face of global change. We did not specifically investigate the effect of river network structure on fish diversity but rather demonstrated the relationship between area and fish diversity through area sampling of river distributions on a global scale. Our results indirectly suggested that an increased protected catchment area could benefit a greater number of fish species.

We sought to test the rationale for integrating fishes, mammals, and birds into a unified analysis process. Although our results showed that all 3 groups followed the SAR, suggesting that the data obtained from area sampling were consistent with basic ecological laws, it is important to note that the generation of the SAR involved the use of random combinations of samples to create an area gradient (Galiana et al., 2022). The observed power function relationship may be a fixed pattern resulting from this particular method. To further test the power function relationship independent of the random combination method, we used the same analytical process to examine the relationship between river length and area. We found that river length increased linearly with area rather than following a power function relationship. This suggested that the observed fixed pattern of change was not a result of the random combination method. In addition, we found no difference in the rate of change in the linear relationship between river length and area based on the random combination method and the expanded area sampling method. This further suggested that the SAR of fishes was independent of the random combination method.

The co‐occurrence of groups with high species richness is a guiding principle for ABC strategies. High‐value areas where different conservation objectives co‐occur are considered the highest priority areas (Di Marco et al., 2016; Kitching, 2000). However, our results showed that co‐occurrence trends of high species richness were not as common across different target groups as we thought they would be (Figure 4). Spatially, the high species richness values of the different groups were not always clustered (Figure 4). This indicated that the ability of ABC to protect different groups is questionable. Some groups can be managed uniformly, but there will be some groups with high species richness values that are outliers, and these groups, including fishes, are essential for maintaining biodiversity integrity (Dudgeon et al., 2006; McFadden et al., 2023). Thus, they need special protection. Fish were outliers, but outliers need to be redefined in other combinations. We found that multitarget conservation did not necessarily cover all conservation targets, and these outliers need to be scientifically assessed and to have separate conservation policies developed for them to maintain biodiversity integrity.

One should exercise caution when generalizing our results to unsampled areas. The global representation of the sampled data needs improvement. In the process of data processing, the absence of fish data directly affected the spatial range that could be represented in our study. Therefore, it is feasible to further strengthen global cooperation to increase the intensity of field surveys of fishes to test the universality of our results for the tropics and other unsampled areas. It should also be a reminder that due to biased field surveys, species richness maps based on the IUCN assessments may be uncertain and require further global cooperation. We included only a single component of biodiversity (species richness), whereas genetic diversity, functional diversity, and interaction diversity are also important components, which need further research. We focused on the rational allocation of protected areas to enhance biodiversity conservation outcomes. However, the allocation of protected areas must be based on consideration of both ecological and social conditions, which is a key point that future research should also address.

## Supporting information



Supporting Information
Additional supporting information may be found in the online version of the article at the publisher's website.

## Data Availability

The data and script supporting our findings are available from https://doi.org/10.5281/zenodo.13923523 and https://doi.org/10.5281/zenodo.13923531, respectively, and in the Supporting Information of this article.
