# Supplementary material for: Potential for synergistic conservation through area‐based strategies
Source: Conserv Biol. 2025 Feb 13;39(4):e14447. doi: 10.1111/cobi.14447 (PMC12309653; doi:10.1111/cobi.14447)
Supplement: Supplementary file 1 — Supporting Information Additional supporting information may be found in the online version of the article at the publisher's website. [file COBI-39-e14447-s001.zip › Supporting Information.docx]

**Supporting Information**

Appendix S1-8 were stored on a publicly accessible library.

**Spatial autocorrelation analysis within group**

$Global I= \frac{\sum_{i=1}^{n} \sum_{j=1}^{n} W_{ij}\left( X_{i}-\bar{X} \right)\left( X_{j}-\bar{X} \right)}{S^{2}\sum_{i=1}^{n} \sum_{j=1}^{n} W_{ij}}$ Appendix S9

$S^{2}=\frac{1}{n}\sum_{i=1}^{n} \left( X_{i}-\bar{X}^{2} \right)$ Appendix S10

$\bar{X}=\frac{1}{n}\sum_{i=1}^{n} X_{i}$ Appendix S11

n is the number of hexagons;$X_{i}$ and $X_{j}$ represent the species richness of hexagon i and j respectively; $\bar{X}$ refers to the average of species richness of all hexagons; and $W_{ij}$ indicates the spatial weight matrix.

The significance of the Global I (GI) index can be tested used the Z-score.

$Z=\left( \frac{I-E\left( I \right)}{\sqrt{Var\left( I \right)}} \right)$ Appendix S12

The range of the GI index is from −1 to 1. If the value of the GI index is greater than 0, this implies a positive spatial correlation and obvious spatial clusters of cells with higher attribute values or lower attribute values. When the value of the GI index nears 1, greater spatial agglomeration is present. Inversely, when the GI index is less than 0, this indicates negative spatial correlation and an obvious spatial difference in the attribution values between the cells and their adjacent cells; when the GI index nears 0, this reflects the a spatially random expression of the pattern, that does not exhibit qualities of spatial autocorrelation.

$Local I=Z_{i}\sum_{j=1}^{n} W_{ij}Z_{j}$ Appendix S13

$Z_{i}=\left( X_{i}-\bar{X} \right)/{\frac{1}{n}}\sum_{i=1}^{n} \left( X_{i}-\bar{X} \right)^{2}$ Appendix S14

$Z_{j}=\left( X_{j}-\bar{X} \right)/{\frac{1}{n}}\sum_{j=1}^{n} \left( X_{j}-\bar{X} \right)^{2}$ Appendix S15

where $Z_{i}$ and $Z_{j}$ are the standardized observed values of city i and j.

If Local I (LI) is a positive value, this shows that species richness has a spatial association and resemble the values of the surrounding hexagons, namely, HH clustering or LL clustering. HH cluster means the occurrence of High-High associations-the clustering of similar high observation values. LL cluster displays low values surrounded by low neighboring values. If LI is a negative value, this indicates that the hexagon i has a very different species richness value than its surrounding hexagons, namely, HL or LH outliers. HL cluster and LH cluster indicate spatial association of dissimilar values: high values surrounded by low values for the former, and low values surrounded by high neighboring values for the latter. The significance of the LI index can be tested by means of a Z-score.

**Spatial correlation analysis cross group**

Spatial lag model:

$Y=\alpha+\rho WY+\beta X+\varepsilon$ Appendix S16

where Y is a vector of dependent variables, X is a matrix of explanatory variables, W is the spatial weight matrix, and WY is a vector of spatial lag dependent variable. $\rho$ is a spatial regression coefficient that reflects the spatial dependence of the sample observations. $\beta$ is a vector of parameters and $\varepsilon$ is a normally distributed disturbance term with a diagonal covariance matrix.

Spatial error model:

$Y=\alpha+\beta X+\varepsilon$ Appendix S17

$\varepsilon=\lambda W_{\varepsilon}+\mu$ Appendix S18

where Y is a vector of dependent variables, X is a matrix of explanatory variables, W is the spatial weight matrix, $\beta$ is a vector of parameters.

where $\mu$ is an i.i.d residual. The parameter $\lambda$ is the spatial autoregressive coefficient that reflects the influences of the residuals of adjacent area on the residuals of the local area.


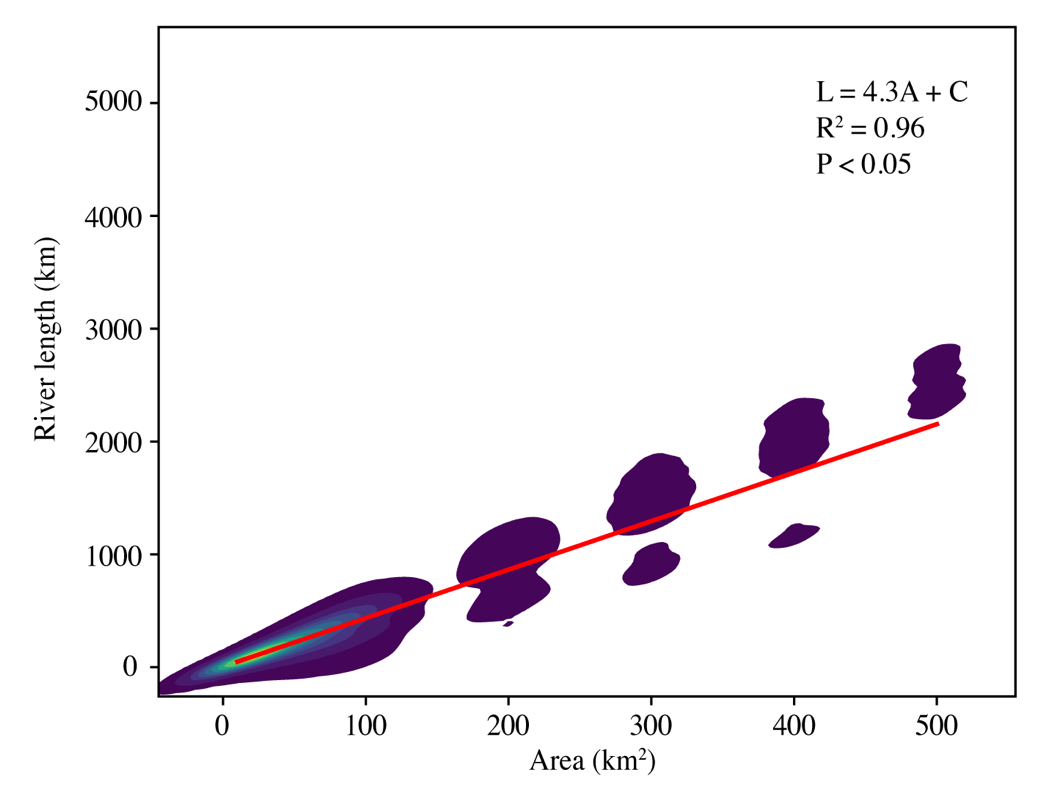


Appendix S19 Relationship between sampling area and river length, real area growth gradient (10-500 km^2^).


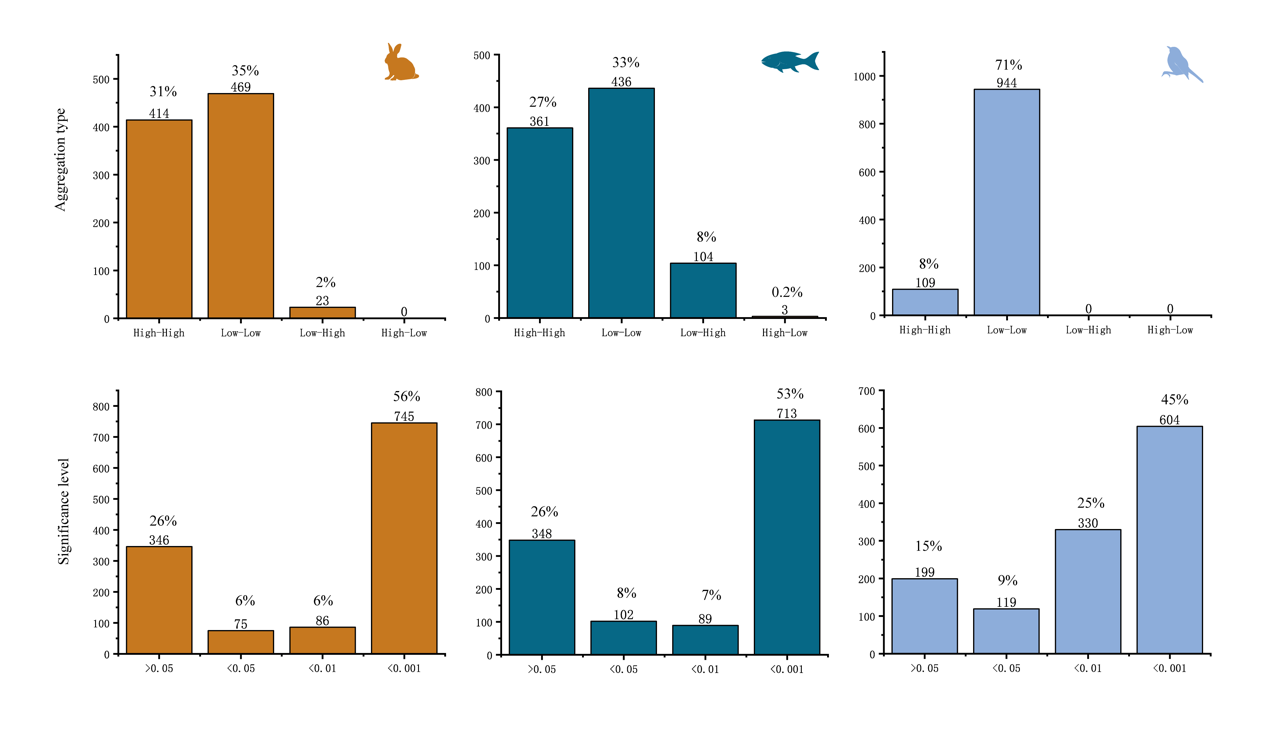


Appendix S20 Local Moran’ I analysis results.

Appendix S21 OLS results and Lagrange Multiplier (LM) test/ robust LM test of mammals-birds group.

| Groups | Units | LM-lag | LM-error | Robust  LM-lag | Robust  LM-error | R^2^ |
| --- | --- | --- | --- | --- | --- | --- |
| Mammals-birds | 100 | 766.35* | 3662.60* | 6.59* | 2902.83* | 0.10* |
| Mammals-birds | 200 | 430.17* | 1788.30* | 11.43* | 1369.55* | 0.14* |
| Mammals-birds | 300 | 196.47* | 1404.64* | 50.63* | 1258.80* | 0.12* |
| Mammals-birds | 400 | 194.87* | 1376.53* | 37.48* | 1219.14* | 0.19* |
| Mammals-birds | 500 | 164.01* | 1201.98* | 43.09* | 1081.06* | 0.21* |

Appendix S22 OLS results and Lagrange Multiplier (LM) test/ robust LM test of fish-mammals group.

| Groups | Units | LM-lag | LM-error | Robust  LM-lag | Robust  LM-error | R^2^ |
| --- | --- | --- | --- | --- | --- | --- |
| Fish-mammals | 100 | 1384.92* | 2033.82* | 21.56* | 670.47* | 0.09* |
| Fish-mammals | 200 | 885.07* | 1209.81* | 11.37* | 336.11* | 0.08* |
| Fish-mammals | 300 | 773.62* | 1048.90* | 18.16* | 293.44* | 0.09* |
| Fish-mammals | 400 | 719.73* | 979.95* | 17.82* | 278.04* | 0.07* |
| Fish-mammals | 500 | 484.85* | 618.72* | 10.43* | 144.31* | 0.04* |

Appendix S23 OLS results and Lagrange Multiplier (LM) test/ robust LM test of fish-birds group.

| Groups | Units | LM-lag | LM-error | Robust  LM-lag | Robust  LM-error | R^2^ |
| --- | --- | --- | --- | --- | --- | --- |
| Fish-birds | 100 | 1679.37* | 2357.26* | 4.67* | 682.57* | 0.01* |
| Fish-birds | 200 | 996.37* | 1301.66* | 3.18* | 308.47* | 0.01* |
| Fish-birds | 300 | 867.90* | 1142.54* | 1.37* | 276.01* | 0.01* |
| Fish-birds | 400 | 779.62* | 1040.71* | 4.77 | 265.85* | 0.02* |
| Fish-birds | 500 | 491.82* | 614.74* | 3.28 | 126.20* | 0.02* |

Appendix S24 Spatial error model results of mammals-birds group.

| Groups | Units | R^2^ | Coefficient of independent variable (β) | λ |
| --- | --- | --- | --- | --- |
| Mammals-birds | 100 | 0.75* | 0.20* | 0.87* |
| Mammals-birds | 200 | 0.74* | 0.23* | 0.85* |
| Mammals-birds | 300 | 0.76* | 0.28* | 0.88* |
| Mammals-birds | 400 | 0.80* | 0.26* | 0.89* |
| Mammals-birds | 500 | 0.80* | 0.27* | 0.88* |

Appendix S25 Spatial error model results of fish-mammals group.

| Groups | Units | R^2^ | Coefficient of independent variable  (β) | λ |
| --- | --- | --- | --- | --- |
| Fish-mammals | 100 | 0.56* | 0.10* | 0.79* |
| Fish-mammals | 200 | 0.55* | 0.12* | 0.76* |
| Fish-mammals | 300 | 0.52* | 0.14* | 0.76* |
| Fish-mammals | 400 | 0.54* | 0.13* | 0.76* |
| Fish-mammals | 500 | 0.45* | 0.11* | 0.73* |

Appendix S26 Spatial error model results of fish-birds group.

| Groups | Units | R^2^ | Coefficient of independent variable (β) | λ |
| --- | --- | --- | --- | --- |
| Fish-birds | 100 | 0.56 | 0.003 | 0.80* |
| Fish-birds | 200 | 0.54 | -0.001 | 0.77* |
| Fish-birds | 300 | 0.51 | 0.02 | 0.77* |
| Fish-birds | 400 | 0.53 | 0.002 | 0.76* |
| Fish-birds | 500 | 0.44 | 0.02 | 0.74* |

Appendix S27 Spatial lag model results of mammals-birds group.

| Groups | Units | R^2^ | Coefficient of independent variable (β) | ρ |
| --- | --- | --- | --- | --- |
| Mammals-birds | 100 | 0.41* | 0.08* | 0.48* |
| Mammals-birds | 200 | 0.40* | 0.09* | 0.47* |
| Mammals-birds | 300 | 0.29* | 0.12* | 0.42* |
| Mammals-birds | 400 | 0.36* | 0.11* | 0.40* |
| Mammals-birds | 500 | 0.38* | 0.13* | 0.41* |

Appendix S28 Spatial lag model results of fish-mammals group.

| Groups | Units | R^2^ | Coefficient of independent variable (β) | ρ |
| --- | --- | --- | --- | --- |
| Fish-mammals | 100 | 0.52* | 0.09* | 0.71* |
| Fish-mammals | 200 | 0.52* | 0.10* | 0.69* |
| Fish-mammals | 300 | 0.50* | 0.14* | 0.68* |
| Fish-mammals | 400 | 0.53* | 0.12* | 0.69* |
| Fish-mammals | 500 | 0.43* | 0.11* | 0.67* |

Appendix S29 Spatial lag model results of fish-birds group.

| Groups | Units | R^2^ | Coefficient of independent variable (β) | ρ |
| --- | --- | --- | --- | --- |
| Fish-birds | 100 | 0.51 | 0.002 | 0.73* |
| Fish-birds | 200 | 0.50 | -0.001 | 0.71* |
| Fish-birds | 300 | 0.47 | 0.010 | 0.72* |
| Fish-birds | 400 | 0.50 | 0.000 | 0.71* |
| Fish-birds | 500 | 0.41 | 0.005 | 0.69* |
